# IoT-Chain and Monitoring-Chain Using Multilevel Blockchain for IoT Security

**DOI:** 10.3390/s22218271

**Published:** 2022-10-28

**Authors:** Dongjun Na, Sejin Park

**Affiliations:** Department of Computer Engineering, Keimyung University, Daegu 1095, Korea

**Keywords:** Internet of Things, multilevel blockchain, lightweight, data reliability, privacy protection

## Abstract

In general, the Internet of Things (IoT) relies on centralized servers due to limited computing power and storage capacity. These server-based architectures have vulnerabilities such as DDoS attacks, single-point errors, and data forgery, and cannot guarantee stability and reliability. Blockchain technology can guarantee reliability and stability with a P2P network-based consensus algorithm and distributed ledger technology. However, it requires the high storage capacity of the existing blockchain and the computational power of the consensus algorithm. Therefore, blockchain nodes for IoT data management are maintained through an external cloud, an edge node. As a result, the vulnerability of the existing centralized structure cannot be guaranteed, and reliability cannot be guaranteed in the process of storing IoT data on the blockchain. In this paper, we propose a multi-level blockchain structure and consensus algorithm to solve the vulnerability. A multi-level blockchain operates on IoT devices, and there is an IoT chain layer that stores sensor data to ensure reliability. In addition, there is a hyperledger fabric-based monitoring chain layer that operates the access control for the metadata and data of the IoT chain to lighten the weight. We propose an export consensus method between the two blockchains, the Schnorr signature method, and a random-based lightweight consensus algorithm within the IoT-Chain. Experiments to measure the blockchain size, propagation time, consensus delay time, and transactions per second (TPS) were conducted using IoT. The blockchain did not exceed a certain size, and the delay time was reduced by 96% to 99% on average compared to the existing consensus algorithm. In the throughput tests, the maximum was 1701 TPS and the minimum was 1024 TPS.

## 1. Introduction

The use of the Internet of Things (IoT) has been increasing, and it is applied to various services in numerous fields [1], such as healthcare, energy, and smart homes. The anticipated IoT device growth is also on the rise. According to Exploding Topics [2], there are over 700 million IoT devices installed worldwide. That number is expected to over 30 billion by 2025. As IoT devices in homes, industrial environments, transportation networks, and elsewhere continue to proliferate, so does the attack surface for malicious IoT network attackers.

**Figure 1:**
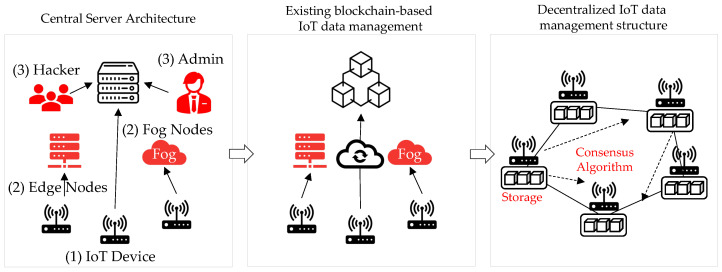
Comparison of IoT data management methods according to architecture (red indicates vulnerability).

The IoT attack activity in 2020 dramatically surpassed the combined volume of IoT activity observed by IBM Security X-Force [3] in 2019. The IoT network is a centralized structure, and all IoT devices mainly use a structure that is linked to the central cloud, or data are stored in the cloud storage using fog and edge computing technologies. Owing to the centralized structure, the central server processes all data, and security vulnerabilities exist owing to the risk of data forgery and falsification, along with attacks on the central processing system, such as distributed denial of service (DDoS) [4] attacks. Depicted in Figure 1 is the centralization problem of existing IoT servers.

**Vulnerabilities in centralized server architecture (1)** Problems owing to IoT device performance limitations: The data measured by the sensor are transmitted to the server. The performance is worse than those of the existing PCs and servers, so there exists a limit [5] to applying existing security vulnerability solutions. Therefore, the Mirai botnet [6] may become a device that executes a server DDoS attack owing to malware infection through firmware updates.

**Vulnerabilities in centralized server architecture (2)** Vulnerabilities owing to fog and edge nodes: These nodes receive real-time data from IoT devices and provide temporary data storage until the necessary data are sent to the cloud. If the fog node is subjected to a security attack [7], it is possible that IoT-generated data may be forged or tampered with. Furthermore, the edge node computes and processes data in edge computing close to the data collection source and sends the data to the cloud. It serves to transmit data. Edge nodes also exhibit a problem in that data are forged and stolen when subjected to a security attack [8].

**Vulnerabilities in centralized server architecture (3)** Security weakness owing to centralized structure: The administrator is responsible for the performance and management of data stored in the central server. Data may be forged or falsified because of the authority of the administrator of the centralized server [9]. Moreover, owing to attacks by hackers and infected devices, the centralized server affects all systems if one server is attacked as a result of a single point of failure [10].

### 1.1. Motivation

Blockchain technology is used to solve the problem of centralized structures [11]. Blockchain is a distributed system that shares information with all non-central participants and distributes data to all network nodes using a peer-to-peer (P2P) network. The problem of data forgery and falsification of existing centralized servers can be solved by allowing nodes in all networks to store the same data. In addition, by securing the reliability of data through an inter-node consensus algorithm, the security problem of IoT devices can be solved with the blockchain function.

However, due to the limitations of blockchain technology, there is a limit to operation in IoT devices, and high-performance external cloud and edge computing technologies are used. This guarantees the immutability of the stored data, but it goes through multiple gateways and nodes during the storage process and cannot guarantee the reliability of the data. **Therefore, the blockchain-based IoT data management method cannot achieve decentralization and cannot solve the limitations of the existing centralized structure.**

### 1.2. Challenges

Blockchain technology can solve the security vulnerabilities of IoT devices, but it has the following limitations.

#### 1.2.1. Capacity Requirement

For an IoT device to become a full node that maintains the blockchain in the blockchain network, all of the blockchains must be stored in storage. However, IoT devices are not suitable for participating as full nodes in blockchain networks because of their low storage capacities.

#### 1.2.2. Consensus Requirement

The consensus algorithm for block generation uses proof of work (PoW) [12], which requires a high amount of CPU operation, or practical Byzantine fault tolerance (PBFT) [13], which requires a certain amount of network communication. For IoT devices, a consensus algorithm that requires substantial computation is not suitable owing to its low performance [14], and a consensus algorithm that requires substantial communication in an IoT network with many nodes is not effective, particularly when using a wireless network.

#### 1.2.3. Data Privacy

Blockchain discloses data transparently, as all nodes have the same blockchain. As IoT data are increasingly used, personal data are also created and managed; thus, data privacy must be guaranteed.

### 1.3. Contribution

We propose an IoT-operable blockchain that overcomes the limitations of the blockchain that cannot guarantee privacy because of the storage space requirement owing to the increase in the blockchain length, the amount of computation and network communication of the consensus algorithm, and data transparency. To this end, we present a multilevel blockchain structure consisting of the IoT-Chain, which is a lightweight blockchain that operates on IoT devices and is pluggable into Hyperledger Fabric or Ethereum, and the Monitoring-Chain, which is implemented based on Hyperledger Fabric.

The IoT-Chain, which is a blockchain that operates on IoT devices, and a monitoring chain to solve the performance limitations of devices that operate nodes of the IoT-Chain, are configured. In addition, by applying the lightweight consensus algorithm of IoT-Chain and the process of periodically distributing and storing IoT-Chain and storing only metadata after verification through consensus of all nodes, a reliable lightweight technique is applied to centralize existing IoT device data management. The limitations of the existing server structure can be solved through blockchain.

#### 1.3.1. Capacity Requirement

We propose the IoT-Chain and Monitoring-Chain to lighten the blockchain. Blocks are created in the IoT-Chain through a consensus method in which all nodes using the Schnorr signature participate and verify the hash value of the last block to ensure the reliability between the two blockchains that can be operated on IoT devices. After distributing and storing the chain in a distributed file system, only the address value is embedded into the Monitoring-Chain, following which the blockchain of the IoT-Chain is initialized so that the blockchain capacity stored in the IoT-Chain does not exceed a specific size. The blockchain can be maintained even in IoT devices with limitations.

#### 1.3.2. Consensus Requirement

We propose a block generation consensus algorithm that overcomes the limitations of the IoT device CPU operation and network communication volume. By using the verifiable random function (VRF) [15] and public key infrastructure (PKI) [16], we present a method to select a leader node for generating a block by determining verifiable random values.

#### 1.3.3. Data Privacy

After distributing the IoT-Chain and embedding the returned address in the Monitoring-Chain, it was stored in the Monitoring-Chain network that is constructed based on Hyperledger Fabric [17], which is a permissioned blockchain, and it is only accessed by authorized users through smart contracts [18]. The data can be accessed to ensure the privacy of IoT data. The proposed blockchain structure was implemented and registered as an open source program [19]. It was verified that it can operate in IoT devices. Subsequent papers will consist of research relating to the proposed blockchain structure, and descriptions of the constructed blockchain module and system, architecture, analysis, experiments, and results.

## 2. Related Work

We compare existing lightweight blockchains in Table 1. The comparison is based on the node’s operating location, TPS, storage overhead, amount of computation, whether access control is supported, scalability, and whether off-chain storage is used. The amounts of computation were divided into PoW (high), PoS (mid), and PBFT (low) series based on the consensus algorithm. Sensor-chain [20] was proposed as a lightweight solution to use IoT devices appropriately for blockchain. Spatial blockchain [21], which divides the blockchain into spatial units, and the migration manager function [22], were used when migrating according to time. Each blockchain owns the blockchain according to the space, and as time passes, the contents of all blockchains are summarized in one block, following which the blockchain size is reduced by starting from that block. However, data loss may occur by summarizing the data that are collected from IoT devices as data such as averages, and only simple data such as sensor values can be used.

IOTA [23] is a cryptocurrency platform that is designed to apply blockchain to the IoT. It uses the proprietary tangle algorithm of the blockchain to reduce the blockchain size and to make it suitable. However, owing to the semicentralized form that cannot achieve complete decentralization of the IoT of the blockchain, the nodes that store a large amount of data may be attacked, and there are vulnerabilities in the hacking methods, such as centralized attacks. Furthermore, it is not suitable for IoT devices with low computing power using PoW as a consensus algorithm.

In BPIIoT [24] and Fusion-Chain [25], the InterPlanetary File System (IPFS) that utilizes a distributed hash table (DHT) for lightweight functions is used to store transactions, which are sensor data, in blocks, and to distribute and store blocks and accessible addresses. It uses a method to store only the values. However, in this study, the IPFS is not a method for distributing and storing the blocks to be stored. The blockchain is distributed and stored after the consensus of all nodes at a specific cycle, and then exported to another blockchain, and the blockchain is restarted.

EdgeChain [26], using edge computing, applies a credit-based resource management system to control all IoT devices from the edge server. Moreover, it stores all activations and transactions created by the IoT devices, and audits the data resources of the IoT devices through records. In the case of BlockEdge, blockchain is used to audit the different processes involved in industrial IoT applications.

HyperLoRa [27] detects tampering of IoT data using Hyperledger Fabric. Certain studies [28] have also used a smart grid network and permissioned blockchain.

BLA [29] uses blockchain in a fog-based IoV environment, and the Group Signature and Authentication Scheme for Blockchain-Based Mobile-Edge Computing [30] was used to authenticate new users in multi-access edge computing. This enables local authentication without the need for authentication from the cloud; thus, it is possible to reduce the latency required for verification in edge computing.

However, in all of the above methods, the blockchain does not work in IoT devices, and vulnerabilities exist, as illustrated in Figure 1. Regarding blockchains based on sharding, OmniLedger [31], RapidChain [32], and RepChain [33] are limited to cryptocurrency applications. RepChain also defines the reputation by using the trust and activeness of validators, based on their decisions on the list of transactions. RepChain uses the accumulated reputation values for balanced sharding and leader selection. However, when the leader node is selected through the accumulated values of the reputation, the randomness is reduced. In this study, a leader node was randomly selected using VRF.

HomeChain [34], which is a novel attribute-based access control scheme for IoT systems [35], and Fabric-IoT [36], have been used for the access control of IoT devices based on blockchain. In this study, the access control function for the user is implemented for security by using the smart contract of the blockchain, but forgery and falsification of the sensor data storage generated by the IoT device mean that stability is not guaranteed. Studies on consensus algorithms have been conducted, including PoBT [37], Algorand [38], and Tendermint [39].

PoBT consists of a structure that is suitable for IoT scalability and performance based on the consensus structure of Hyperledger Fabric, and Algorand is a VRF-based consensus algorithm. Tendermint is a PBFT-based consensus algorithm. In this study, although Hyperledger Fabric is used, a new blockchain that operates on IoT devices is proposed. A random-based consensus algorithm is suitable for IoT performance. Unlike PBFT, a consensus method and lightweight method that are suitable for network traffic and sensor generation speed are proposed. Moreover, as opposed to Algorand, this study presents a blockchain that is suitable for IoT devices by selecting a consensus leader node based on the size of a value without using VRF to form a committee.

Reference [40] proposed a consensus algorithm that improves the performance of the routing algorithm. A lightweight blockchain-based secure routing algorithm has been proposed to address the challenges [41] of swarm unmanned aircraft system (UAS) networking, and a proof of traffic consensus algorithm that reduces passive broadcasts is used.

Reference [42] proposed a consensus construction with high verification efficiency with base stations for cellular network connection. Unlike the above paper, in this paper, we propose an efficient group authentication method based on the consensus algorithm and Schnorr signature based on lightweight communication in IoT-Chain.

Reference [43] proposed a blockchain that requires minimal hardware and software performance. To this end, we propose a witness protocol, which can maintain a blockchain with minimal hardware, and does not require additional equipment or access to a centralized network. In contrast, the blockchain in this paper is a multi-level blockchain configuration so that blockchain nodes can participate in the maintenance of the blockchain and participate in the consensus algorithm and data verification process.

## 3. Background

### 3.1. Hyperledger Fabric

Hyperledger Fabric [17] is a private blockchain that provides network security and distributed ledger technology with scalability, confidentiality, and improved performance. Furthermore, as network roles are assigned for each node type in Hyperledger Fabric, network concurrency and parallelism are provided, which offers the advantage of high TPS compared to public blockchain platforms. In this study, the monitoring chain was implemented with Hyperledger Fabric, and the chaincode of Hyperledger Fabric was used to monitor the data generated by the IoT sensors and to implement the access control function to check the user’s authority when accessing the data.

### 3.2. InterPlanetary File System

The IPFS [44] distributes files such as photos, texts, and videos on the Internet and uses a unique hash value to load the distributed data rapidly. High-capacity files can be loaded quickly and efficiently, and by using a unique hash value, the existence of duplicate files can be known for efficient file storage. The nodes participating in the IPFS network manage the hash table independently and store data without a server. This provides a method of mapping filenames to values in the hash table that is held by each node without using a central server through the DHT. The DHT can suppress the load on the network, and enables the fast and accurate retrieval of files in the network. The IPFS is content addressed, where the file itself acts as an address. If the file name (CID) is found in the DHT through data, the node that has the distributed fragment of the file is found and the file is loaded. When distributing files, the IPFS converts all files on the network into the Merkle-DAG format. For each node, the CID, which is a hash of the node content, is used for Merkle-DAG. By using Merkle-DAG, the IPFS can address content, and prevent tampering and duplication. By using these functions, the IPFS distributes and stores the blockchain for every round of the IoT-Chain and returns a hash value to the monitoring chain. Data can be accessed on the IoT-Chain through the hash value that is stored in the Monitoring-Chain.

### 3.3. Schnorr Signature

The Schnorr signature [45] is similar to the method of extracting a public key from a single number (e.g., private key) in the elliptic curve digital signature algorithm (ECDSA) [46], but has the characteristic of linearity. The ECDSA involves many heavy operations, such as modular inverse and points multiplication, in the calculation process, whereas Schnorr has relatively few heavy operations in comparison. Schnorr signatures also enable collective signatures that combine the signatures of multiple signers into a single signature. As a result, N signatures can be verified once instead of N times, and the size of the signature is reduced. In this study, the Schnorr signature is used when signing following the verification of all nodes.

### 3.4. Verifiable Random Function

The VRF [15] is a function that outputs a verifiable pseudo-random value for the input. The VRF generates a unique and verifiable pseudorandom number Y for input X when the key pairs VK and SK are fixed. The VRF is similar to the digital signature method in that the output can be verified using a separate verification key. However, the digital signature differs from the VRF function in that multiple valid signatures exist for one input, and the output value is not sufficiently random to satisfy the pseudorandom number condition. In this study, it is used to select a leader node in the consensus process of the IoT-Chain that can be operated in IoT devices. All IoT-Chain nodes that create a transaction execute the VRF function, include the returned result value in the transaction, and send it to the leader node. The leader node selects the node with the largest VRF value as the next leader node and creates a block to propagate to all of the nodes.

## 4. Architecture

The proposed architecture is a multi-level blockchain structure consisting of an IoT-Chain network that can only be configured with IoT devices, and a monitoring chain to increase the size of the IoT-Chain and solve the limitations of controlling access to data. Through a multi-level blockchain structure, decentralized data management is possible only with IoT devices, and blocks are verified using consensus that does not require a computational load through a PBFT-based consensus algorithm in the IoT chain. In addition, when exporting blockchain from IoT-Chain to Monitoring-Chain, signatures of all IoT-Chain nodes are required during the consensus process, and network communication must be guaranteed not to be lost during consensus. Therefore, by using the Schnorr signature method, the capacity of the signature is reduced in the consensus process to reduce the message size during the consensus of the export process, and there are nodes that deliver only the external monitoring chain and metadata through the Exporter node. Forgery and falsification of the node’s data can be guaranteed through the Schnorr signature, which contains the compressed signatures of all nodes. The following describes the overall architecture of the structure proposed in this paper, the structure and operation of the IoT-Chain, and the Monitoring-Chain.

### 4.1. System Design

**Figure 2:**
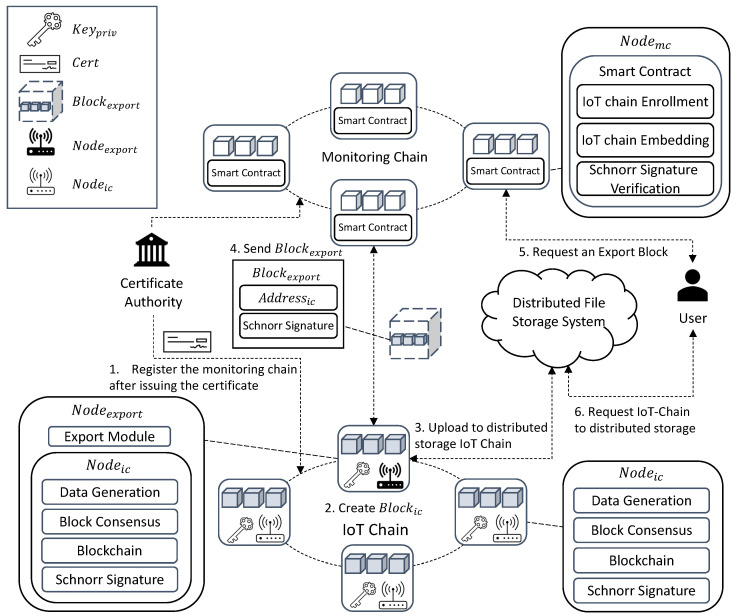
System Architecture: (**1**) All $Node_{ic}$ register their identity through CA and then participate in the network and register the IC by storing the Genesis Block hash value in the MC. All IoT-Chain nodes must obtain a key through CA and register a certificate for the public key. In (**2**) $Network_{ic}$, Nodeic generates the sensor data, $Block_{ic}$ is generated, and the length of the IC increases. (**3**) To maintain the IC at no more than a certain size (storage capacity of IoT device), $Node_{export}$ is all $BH_{last}$ of all $Node_{ic}$. By starting a consensus to verify that all $Node_{ic}$ have the same IC, $Sig_{schnorr}$ and $Key_{schnorr}$ are created. (**4**) $Node_{export}$ uploads IC to DFS and returns $Address_{ic}$ and $Block_{export}$ with $Sig_{schnorr}$, and $BH_{last}$ is created and passed to $Network_{mc}$. $Node_{mc}$ requests $Key_{pub}$ from CA, creates $Key_{schnorr}$, validates $Sig_{schnorr}$, and if verified, $Block_{export}$ is MC, which is saved and embedded. (**5**) For user access control, the smart contract verifies the access rights of the user, and if verified, it allows access to $Block_{export}$ stored in the MC. (**6**) The user can access the IC stored in the DFS through $Address_{ic}$ stored in $Block_{export}$.

As depicted in Figure 2, $Network_{ic}$ runs on IoT devices and maintains the IC that stores the sensor data. It consists of $Node_{ic}$, $Node_{leader}$, and $Node_{export}$. $Node_{ic}$ generates sensor data to generate TX. $Node_{leader}$ is randomly selected, collects TX generated by the node, generates $Block_{ic}$, and propagates them to all nodes. $Node_{export}$ maintains a routing table for consensus, sets a path to receive the signatures of all nodes, detects a certain size, and verifies that $BH_{last}$ of all $Node_{ic}$ is the same to start an agreement. Every $Node_{ic}$ encrypts $BH_{last}$ via $Key_{priv}$ to create Sig, which is combined with the signatures generated by other $Node_{ic}$ in the consensus path $Sig_{schnorr}$ and adding $Key_{pub}$ to create $Key_{schnorr}$, and can verify the signatures of all nodes with one verification. All $BH_{last}$ are verified to be the same, and $Node_{export}$ uploads IC to DFS and returns $Address_{ic}$ to create $Block_{export}$. $Network_{mc}$ is implemented based on Hyperledger Fabric, receives $Block_{export}$ generated by $Block_{export}$, and sends $Sig_{schnorr}$ through SC to $Key_{schnorr}$. If the decoded value is the same as $BH_{last}$, it is stored in MC, and the IC of all $Node_{ic}$ starts from $Block_{genesis}$ again. In the IoT-Chain, there are nodes that generate transactions and exporter nodes that lighten the IoT-Chain by periodically communicating with a leader node and a monitoring chain that are randomly selected among nodes and generate blocks. In the case of a leader node, a block is created by receiving the broadcast transaction. An exporter node is a node registered in the monitoring chain as a gateway that ensures network communication rather than a general IoT device for communication with the monitoring chain. In addition, stable communication is ensured through the exporter node, preventing data loss during network communication, and it is possible to ensure that all nodes have verified data for the lightweight blockchain through a compressed signature during the consensus process. Table 2 lists the terms used in this study.

### 4.2. Multilevel Blockchain

**Figure 3:**
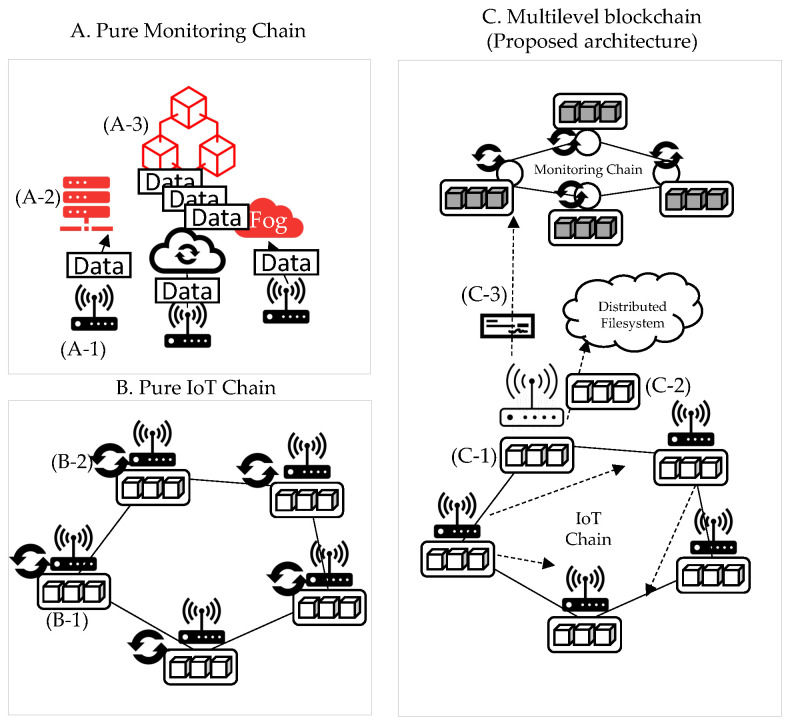
Multilevel blockchain structure.

This section explains why it is composed of a multilevel blockchain. As depicted in Figure 3, we compare the structure (A) consisting of only the IoT chain and the structure (B) consisting of only the monitoring chain. Additionally, in (C), the problem of solving the limitations of the two structures (A, B) in the multilevel blockchain is explained. (A) is a case in which the blockchain is configured with only the monitoring chain in the IoT device’s external network. (A-1) Each IoT device forwards a transaction to the blockchain. For delivery, there are two methods: sending a transaction to the API server and sending it to the blockchain, and sending it to the blockchain. (A-2) is the method of delivery to the server, and data tampering may occur during this process. (A-3) is the process of delivering a transaction directly from an IoT device to a blockchain, and TPS degradation may occur in this process. (B) is a case of maintaining the blockchain with only IoT devices. (B-1) All blockchains are recorded in IoT devices, and even if data are lighter, data size increases. Thus, if it goes above a certain size, IoT devices will not be able to sustain the blockchain due to insufficient storage capacity. In addition, the consensus process is essential for block creation in the blockchain. The consensus algorithm is divided into PoW consensus, which requires CPU operation, and PBFT, which proceeds only through network communication. However, as both consensus algorithms have to be operated on IoT devices as in (B-2), they require CPU computation or high network traffic. (C) is a multi-level blockchain structure to supplement the limitations of (A) and (B). In this structure, IoT devices maintain the IoT-Chain. The monitoring chain supports the IoT-Chain. (C-1) When the storage capacity becomes insufficient, the blockchain from the genesis block to the present is recorded externally (C-2) to the external distributed file system. After recording, the data are backed up in the monitoring chain, and in this process, the export process is performed for data verification. This process is not verified by a single node, but by (C-3) monitoring chain nodes. Therefore, the monitoring chain supports the IoT-Chain in performing data backup and decentralized verification. In addition, the IoT-Chain network is not a private network, but it is necessary to record node information in the monitoring chain for verification in the export process. Therefore, anyone can participate in the network, and the participating sensors are based on P2P communication, ultra-low power, and ultra-low performance hardware, so basic calculations are possible, unlike in a sensor network environment.

### 4.3. IoT-Chain

**Figure 4:**
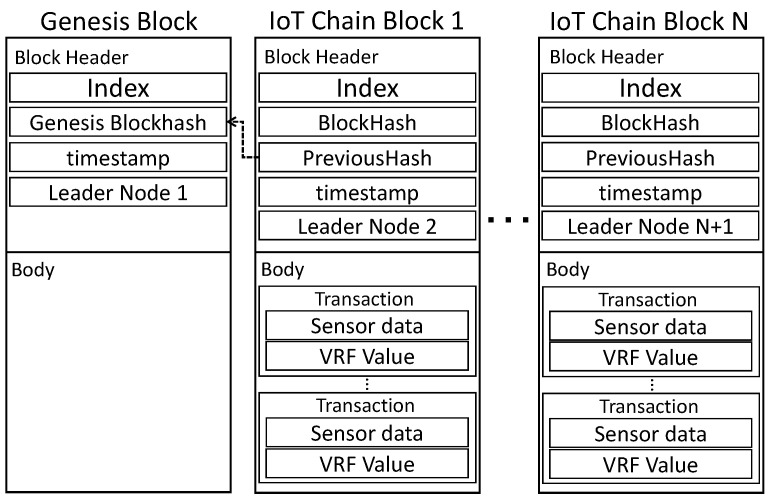
IoT-Chain block: block structure of IoT-Chain.

$Network_{ic}$ creates a block through a lightweight consensus algorithm, and following consensus using $Sig_{schnorr}$ for each round, the address is returned after storing the blockchain in an external distributed file system (DFS). After transferring only the value to $Network_{mc}$, IC is lightened by starting from the Genesis Block. As depicted in Figure 4, presents the block structure of IC. The data in the block are composed of Index, BlockHash, PreviousHash, timestamp, and $Node_{leader}$. $Node_{leader}$ recorded in the block instructs the generation of the next block. TX is created as a node. TX is stored in the body and includes the sensor data, Sig, and $Val_{rand}$.

**Figure 5:**
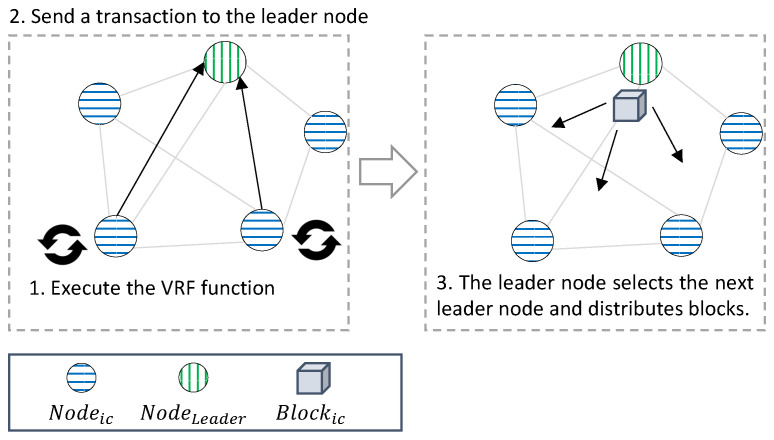
$Block_{ic}$ generation process: (**1**) $Node_{ic}$ executes the VRF function when TX is created, returns $Val_{rand}$, and encrypts data with its own $Key_{priv}$ and Sig. After generation, $Node_{leader}$ is checked to generate the next block in the last block of the blockchain and to send TX. (**2**) The TX structure, Sig, and $Val_{rand}$ of $Node_{leader}$ that received TX are verified. (**3**) If all $Val_{rand}$ are verified, the node with the largest $Val_{rand}$ among the nodes that transmitted TX is selected as the next $Node_{leader}$, recorded in a block, and distributed to all nodes.

Figure 5 depicts the process in which $Node_{ic}$ generates TX and sends TX to $Node_{leader}$, and $Node_{leader}$ generates $Block_{ic}$. The node that generates TX from $Network_{ic}$ executes the VRF and includes the returned value, sensor data, and Sig generated through $Key_{priv}$ in TX, which is sent to $Node_{leader}$.

The $Node_{ic}$ executes VRF every set round and delay, the $Node_{ic}$ with the lowest value is selected as the $Node_{leader}$, and a $Block_{ic}$ is generated from the $Node_{leader}$. In this way, the next $Node_{leader}$ is unpredictable, thus preventing the attack. Round means the cycle to create a $Block_{ic}$, and delay is the time to select a $Node_{leader}$. The selected $Node_{leader}$ creates as many blocks as rounds, and then all nodes execute VRF during delay and broadcast the result to the $Network_{ic}$ in the gossip protocol. After the delay time is over, the node with the lowest value among the nodes is selected as the $Node_{leader}$.

In this paper, to lighten the IoT chain, the blockchain is stored in a distributed file system, and only metadata are stored in the monitoring chain. This process corresponds to the export process, and a monitoring chain and exporter node are required for operation. The monitoring chain enables decentralized verification of the Schnorr signature, the result of the export process, as nodes verify through smart contracts. In addition, the export agreement proceeds in the ring signature [47] method. Therefore, it is necessary to have a node that initiates and terminates consensus, collects it, and sends the result of the consensus to the monitoring chain. The export node is responsible for initiating the export consensus and transferring the generated Schnorr signature to the monitoring chain.

### 4.4. Monitoring-Chain

The monitoring chain serves to support the export process in the IoT-Chain. In the IoT-Chain, after the export process is performed, the “Schnorr signature” is created by combining the signatures of the nodes. If the Schnorr signature is verified with the stored public key, it is verified that all $Node_{ic}$ signed it. However, due to the centralization problem, it cannot be guaranteed that all $Node_{ic}$ signed after a single $Node_{exporter}$ verifies the signature. $Node_{mc}$ is verified through smart contracts, enabling decentralized verification of the export process. Additionally, for decentralized verification through smart contracts, IoT-Chain nodes do not need to know each other, but the monitoring chain needs to know all IoT-Chain nodes. Therefore, in order to participate in the IoT-Chain, $Node_{ic}$ must register the IoT-Chain node in the monitoring chain to participate in the IoT-Chain. This course is essential for lowering storage weight. In addition, the monitoring chain can maintain data and record metadata about the distributed storage of the IoT-Chain. The value maintained in the monitoring chain is an IoT-Chain of a certain length, which is maintained even when all IoT-Chain networks are terminated, and the recorded hash value allows access to the IoT-Chain stored in the distributed file system.

**Figure 6:**
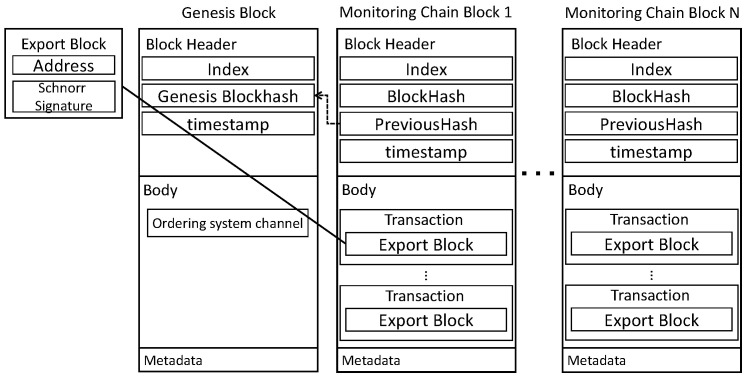
Monitoring-Chain block: Structure of $Block_{mc}$ implemented based on HF. After saving the DFS to lighten the IC, $Address_{ic}$, and $Sig_{schnorr}$, $Sig_{pub}$ is saved. Signature, Index, and TxValidationCode of $Node_{mc}$ are stored in Metadata.

$Network_{mc}$ stores IC in DFS every round in $Network_{ic}$ and returns $Address_{ic}$ and $Sig_{schnorr}$. $BH_{last}$ receives $Block_{export}$ and stores it in MC. Depicted in Figure 6 is the block structure of MC. The BlockHeader includes Index, BlockHash, PreviousHash, and timestamp. TX is stored in the body and $Block_{export}$ is stored in TX.

**Figure 7:**
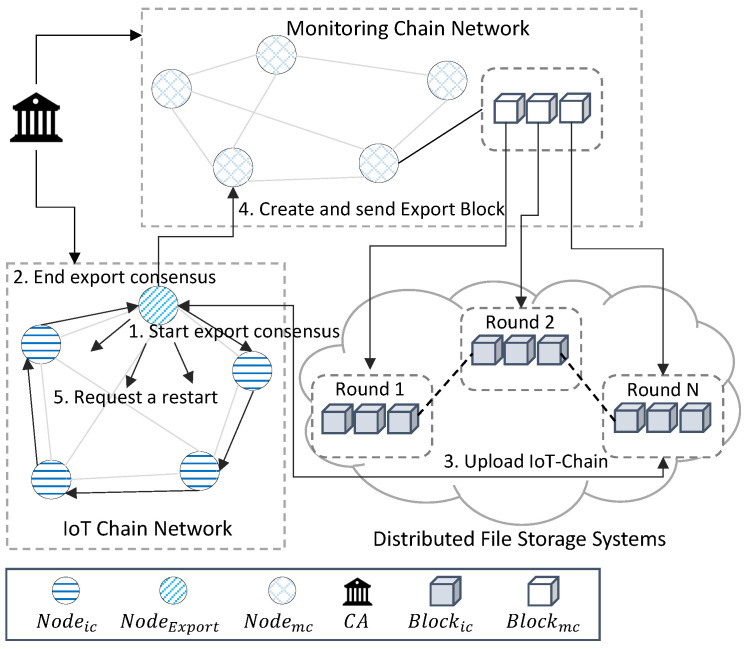
The export process between $Network_{ic}$ and $Network_{mc}$: (**1**) $Node_{export}$ maintains the routing table and agrees to verify that the $BH_{last}$ of all nodes is the same. (**2**) The combined signature of all nodes is sent to $Node_{export}$, and $Key_{pub}$ of $Node_{ic}$ is requested from CA to generate and verify $Key_{schnorr}$. (**3**) IC is saved in the DFS and $Address_{ic}$ is returned. (**4**) $Block_{export}$, including $Address_{ic}$, $Key_{schnorr}$, and $Sig_{schnorr}$, is transferred to $Network_{mc}$ and saved. (**5**) All $Node_{ic}$ of $Network_{ic}$ restart the IC starting with $Block_{genesis}$ with PreviousHash of $BH_{last}$ of IC embedded in MC.

In $Network_{ic}$, $Network_{mc}$ is exported to round, where the IC is larger than a certain size. Depicted in Figure 7 is the process of embedding the IC from $Network_{ic}$ to $Network_{mc}$. $Node_{export}$ acts as a gateway between $Network_{ic}$ and $Network_{mc}$, and among the nodes of $Network_{ic}$, the node with the highest network condition and hardware performance is selected. Alternatively, the routing protocol method of the inferior gateway routing protocol (IGRP), which is a dynamic routing method, maintains the routing table of the consensus process that checks that $BH_{last}$ of all $Node_{ic}$ is the same.

### 4.5. Workflow

This section describes the operation process of the proposed $Network_{ic}$ and $Network_{mc}$. The processes of registering $BH_{genesis}$ to $Network_{mc}$, creating TX and $Block_{ic}$, embedding IC from $Network_{ic}$ to $Network_{mc}$, verifying $Blokc_{export}$ in $Network_{mc}$, and starting over from $Block_{genesis}$ in $Network_{ic}$ are explained in order.

Algorithm 1 is the process of saving the information of $Node_{ic}$ to register IC in $Network_{mc}$. All $Node_{ic}$ sends $BH_{genesis}$ of the same IoT-Chain to MC and stores it through SC. The operation process consists of a process of generating a signature and requesting to register a node, and a process of registering node information by verifying the signature. In the case of signature generation, it runs on the nodes of the IoT chain, and in the case of verification, it operates on the nodes constituting the monitoring chain. To register $Network_{ic}$ in $Network_{mc}$, it is necessary to verify that all $BH_{genesis}$ owned by the node are the same. $Node_{ic}$ encrypts $BH_{genesis}$ through $Key_{priv}$ to create Sig, and creates TX containing Sig and $BH_{genesis}$ to send to $Network_{mc}$. $Node_{mc}$ requests $Key_{pub}$ from CA for Sig included in TX, and all Sig included in TX are sent from $Network_{ic}$. If the decrypted value of $BH_{genesis}$ is the same, the corresponding $BH_{genesis}$ is stored in the MC. Multiple $Network_{ic}$ can be distinguished through the saved $BH_{genesis}$, and $Key_{pub}$ of $Node_{ic}$ can be requested from the CA.
**Algorithm 1** $Network_{ic}$ enrollment.  1:**procedurePROCEDURE**$Node_{ic}:$  2:    **for** $Node_{iot}$ in $Network_{ic}$ **do**  3:        Sig$\leftarrow CreateSig(BH_{genesis},Key_{Priv})$  4:        TX←CreateTx(GenesisBH,Sig)  5:        SendTXtoMC(TX)  6:    **end for**  7:**end procedure**  8:     9:**procedurePROCEDURE**$Node_{mc}:$10:    **for** $Node_{mc}$ in $Network_{mc}$ **do**11:        $Sig,BH_{last}$←GetTX()12:        $Key_{pub}$←CA13:        **if** $VerifySig(Sig,BH_{last},Key_{pub})$ is true **then**14:           $SetState(BH_{last})$15:        **end if**16:    **end for**17:**end procedure**

Algorithm 2 is the process of creating blocks and transactions in the IoT-Chain. The operation process consists of the process of creating a transaction, including the data collected from the IoT device through the sensor, and the process of creating a block by including the transaction in the block after verification. In the case of transaction creation, it operates on the nodes of the IoT chain. In the case of block generation after transaction verification, it is operated by a leader node randomly selected from among the nodes of the IoT chain every block generation round. $Node_{ic}$ generates sensor data and encrypts the sensor data using $Key_{priv}$ for TX generation. The VRF is executed to return $Val_{rand}$ and to transmit TX, including sensor data, Sig, and $Val_{rand}$, to $Node_{leader}$ recorded in the last block. $Node_{leader}$ verifies the values stored in TX with $Key_{pub}$ and has the largest value of $Val_{rand}$. After selecting $Node_{ic}$ as the next $Node_{leader}$, it is recorded in $Block_{ic}$, distributed to $Network_{ic}$, and added to the IC. Unlike $O(n^{2})$, which is the communication amount of the PBFT-based algorithm that achieves consensus through network communication, a leader node is selected through a random function, and the block is verified with the communication amount of O(n). Through the consensus algorithm with a reduced number of messages compared to the PBFT-based consensus algorithm, consensus is possible even in IoT devices with low computational power without using the PoW-based consensus algorithm that requires more computational power.
**Algorithm 2** $Network_{ic}$—$Block_{ic}/TX$ creation.  1:**procedurePROCEDURE**$Node_{ic}:$  2:    Data←GenerateSensorData()  3:    Sig$\leftarrow CreateSig(Data,Key_{Priv})$  4:    $Val_{rand}$←VRF()  5:    TX$\leftarrow CreateTX(Data,Sig,Val_{rand})$  6:    $Block_{last}$←GetLatestBlock()  7:    $Node_{leader}$$\leftarrow Block_{Last}.LeaderNode$  8:    $SendTX(TX,Node_{leader})$  9:**end procedure**10:  11:**procedurePROCEDURE**$Node_{leader}:$12:    TX←ReceviceTX()13:    **if** TXs.Length is count **then**14:        TXs←TX15:        Count(TXs) is not count16:        $Node_{leader}$←GetMaxRandomValueNode(TXs)17:        $Block_{ic}$$\leftarrow CreateBlock(Txs,Node_{leader})$18:        $Broadcast(Block_{ic})$19:    **end if**20:**end procedure**

Algorithm 3 is a lightweight storage process for IC. Nodes in $Network_{ic}$ proceed with “export consensus” to store IC in DFS. After the export consensus process, $Block_{exports}$ with the Schnorr signature is delivered to $Network_{mc}$ through the export node. This process is used to periodically store the IoT chain in a distributed file system and record only metadata for verification in the monitoring chain in order to reduce the capacity of the IoT chain. For verification, it is necessary to start the process of verifying data through the hash value of the last block of the IoT chain. In addition, the final block hash value, which is the verified result, and the signatures of IoT-Chain nodes, must be compressed to generate a Schnorr signature. For operation, the export process of the IoT chain is executed in the Exporter Node. The process of verifying and signing the last block hash value is executed in the nodes of the IoT-Chain. $Block_{ic}$ is created in $Network_{ic}$, the size of the IC increases, and the round exceeds a certain size. Each $Node_{ic}$ initiates a consensus verifying that $BH_{last}$ are all the same for embedding into $Network_{mc}$. Among the nodes, $Node_{expoert}$, which is a pre-selected gateway node based on the network and hardware status, is the route of consensus of all nodes through the routing protocol. $Node_{expoert}$ creates a Sig encrypted with $BH_{last}$ with $Key_{priv}$, creates $Block_{export}$ containing $BH_{last}$ and Sig, and delivers it to the next node on the route. After receiving it, $Node_{ic}$ sends $Sig_{schnorr}$ to $Key_{pub}$, which is verified through the CA. If the $BH_{last}$ returned by creating and decrypting $Key_{schnorr}$ is the same as the $BH_{last}$ of its own IC, the corresponding $BH_{last}$ is encrypted with $Key_{priv}$ to create a Sig, the Sig is combined with a Schnorr signature method, and the Sig is added by $Sig_{schnorr}$ to generate the combined Sig. It is created, passed to the next $Node_{ic}$ in the route, and this is repeated until the next $Node_{ic}$ does not exist. Every node in the $Network_{ic}$ repeats this process, the last $Node_{ic}$ sends a $Block_{export}$ to $Node_{export}$, and $Sig_{schnorr}$ sends a $Block_{export}$ to all nodes in CA. If the same $BH_{last}$ value is returned after decrypting with $Key_{schnorr}$ created by combining $Key_{pub}$, the IC is distributed and stored in the DFS, and subsequently, $Address_{ic}$ is returned. $Node_{export}$ transmits $Block_{export}$ to $Network_{mc}$. When using the existing multi-signature method, a signature size of about O(n) and a number of verifications are required, depending on the number of network nodes. The proposed algorithm compresses the signature using the Schnorr signature method. As a result, the number of validations required for group signatures and the storage capacity of the signatures are reduced by O(1). To create metadata of the IoT-Chain, signatures of all IoT-Chain nodes are required. However, in order to receive the signatures of all nodes, the number of signatures increases as much as the number of nodes, the size of the message also increases, and O(n) signature verification processes exist. In the structure of this paper, verification and verification data transmission are possible in a single signature by compressing the signature using the Schnorr signature.
**Algorithm 3**  $Block_{export}$ creation.  1:**procedurePROCEDURE**$Node_{export}:$  2:    **if** SizeOf(IC) is Maximum **then**  3:        Sig$\leftarrow CreateSig(BH_{last})$  4:        rawRoute←routingTable  5:        Route←CreateSig(rawRoute)  6:        $Block_{export}$$\leftarrow CreateBlock_{export}\left(BH_{Last},Sig,Route\right)$  7:        $StartEmbedding(Block_{Export},Cert,Route)$  8:    **end if**  9:**end procedure**10:  11:**procedurePROCEDURE**$Node_{ic}:$12:    **for** $Node_{ic}$ in $Network_{ic}$ **do**13:        $Block_{export}$←receive()14:        $Keys_{pub}$←CA15:        $Key_{schnorr}$$\leftarrow AggregatePub(Key_{schnorr},Keys_{pub})$16:        **if** $Verification(Block_{export}.Sig_{schnorr},Key_{schnorr})$ is true **then**17:           Sig$\leftarrow CreateSig(BH_{last},Key_{priv})$18:           $Sig_{schnorr}$$\leftarrow Block_{export}.Sig_{schnorr}$19:           $Sig_{schnorr}$$\leftarrow CreateSchnorrSig(Sig_{schnorr},Sig)$20:           $Block_{export}.Sig_{schnorr}$$\leftarrow Sig_{schnorr}$21:        **end if**22:        **if** exist(consensusRoute.Next) is true **then**23:           $Broadcast(Block_{Export},Key_{schnorr},Route)$24:           exist(consensusRoute.Next) is false25:           $Broadcast(Block_{Export},Key_{schnorr},Node_{export})$26:        **end if**27:    **end for**28:**end procedure**

Algorithm 4 is the process of transferring and saving $Block_{export}$ from $Network_{ic}$ to $Network_{mc}$. In this process, after verifying the block hash value made by the nodes of the IoT chain, it is guaranteed that all nodes maintain the same blockchain. After this verification process is performed, the process of transmitting the compressed signature and hash value to the monitoring chain is performed. In addition, the process of verifying the result value delivered to the monitoring chain and storing it in the monitoring chain is performed. The resulting value of the export process is executed by the exporter node and transmitted to the monitoring chain, and the verification process is performed in the monitoring chain node. In $Network_{mc}$, $Sig_{schnorr}$, which is stored in $Block_{export}$ and delivered by $Node_{export}$, is requested by the CA. The returned $Key_{pub}$ is combined and verified, and $Block_{export}$ is stored in the MC. In $Network_{ic}$, the IC is restarted by storing the address in the MC and embedding the IC and then performing the hash operation with the stored $BH_{last}$ and creating the connected block.
**Algorithm 4**  $Block_{export}$ verification.  1:**procedurePROCEDURE**$Node_{export}:$  2:    $Block_{export}$←receive()  3:    $Sig_{schnorr}$$\leftarrow Block_{export}.Sig_{schnorr}$  4:    $Keys_{pub}$←CA  5:    $Key_{schnorr}$$\leftarrow CreateSchnorrPub(Keys_{pub})$  6:    **if** $Verification(Sig_{schnorr},Key_{schnorr})$ is true **then**  7:        $Address_{ic}$←DFS(IC)  8:        $Block_{export}.Address$$\leftarrow Address_{ic}$  9:        $SendBlockToMC(Block_{export})$10:    **end if**11:**end procedure**12:  13:**procedurePROCEDURE**$Node_{mc}:$14:    **for** $Node_{mc}$ in $Network_{mc}$ **do**15:        $Block_{export}$←Receivce()16:        $Keys_{pub}$←CA17:        $Key_{schnorr}$$\leftarrow CreateSchnorrPub(Keys_{pub})$18:        **if** $Verification(Sig_{schnorr},Key_{schnorr})$ is true **then**19:           $PutState(Block_{export})$20:        **end if**21:    **end for**22:**end procedure**23:  24:**procedurePROCEDURE**$Node_{ic}:$25:    $ResetIoTChain(Block_{Export}.BH_{last})$26:**end procedure**

## 5. Security Analysis

In this section, on the lightweight blockchain using the proposed multilevel structure of $Network_{ic}$ and $Network_{mc}$, we analyze the existing security solution application’s limitations owing to the low performance, which is the weakness of existing IoT devices, security vulnerabilities of fog nodes and edge nodes, and whether vulnerabilities caused by data forgery owing to the centralized server structure, single-point errors of the central server, and DDoS attacks can be resolved. The STRIDE threat model technique was applied for the existing IoT blockchain threat analysis.

### 5.1. STRIDE Threat Modeling

**Figure 8:**
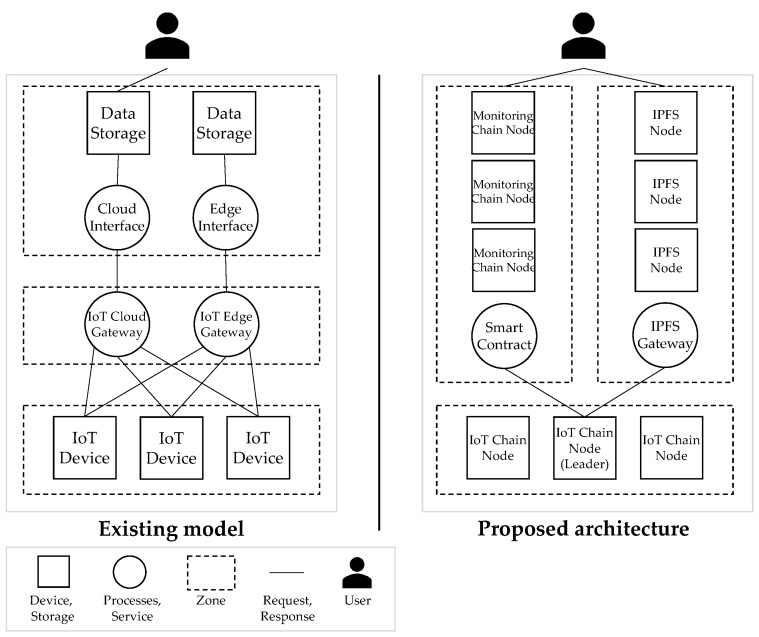
Dataflow diagram in IoT.

According to the Microsoft Threat Modeling Tool threats in Table 3, there are six threats in the STRIDE model: identity spoofing, data tampering, denial, information disclosure, denial of service, and elevation of privilege. We applied the STRIDE model to analyze the threats of existing blockchain-based IoT data management methods and compared them with the proposed structure. Figure 8 shows the data flow of the proposed structure and the existing blockchain-based IoT device data management method. The existing blockchain-based IoT data management method consists of the IoT Device Zone, IoT Filed Gateway Zone, and Cloud/Edge Computing Zone, and the threats of each component were analyzed and compared with those identified in this paper. Table 4 is the analysis of results, and its contents are as follows.

### 5.2. IoT Device Zone Threats

As for the existing IoT device zone threats, there is a method that attempts to log in with spoofed authentication and a threat that exploits it through authentication information. In the structure of this paper, a private key exists for each device and is managed through CA, and all device requests can be used if they are authenticated through a signature. In addition, it is assumed that the private key is not leaked to anyone other than the device and the user.

### 5.3. IoT Flied Gateway Zone Threats

IoT devices are not suitable for application to existing security solutions owing to their low performances. However, the storage requirements of the blockchain and excessive computational amounts of the consensus algorithm will result in traffic problems in the network. Therefore, in the existing blockchain-based IoT data management method, it acts as a client that stores data by sending a transaction through the gateway to a blockchain node operating in an external cloud with a gateway. However, this can lead to information disclosure and data tampering through spoofing attacks on the gateway. In this study, we minimized the use of gateways by using a lightweight blockchain that can operate on IoT devices rather than external blockchain nodes. In addition, during the export process for storage lightening, gateways are randomly selected to prevent attacks.

### 5.4. Cloud and Edge Computing Zone Threats

Existing blockchain-based IoT data management stores data in the cloud and central server. Additionally, the central server is managed by an authorized administrator. Data reliability and integrity cannot be guaranteed due to the possibility of data tampering by privileged administrators and DDoS and SPOF attacks. Through the proposed lightweight blockchain, all IoT devices participate as nodes rather than clients of $Network_{ic}$ to generate $Block_{ic}$ through a consensus algorithm and maintain the IC to ensure data reliability, and the same ledger is held by all $Node_{ic}$, thereby eliminating the possibility of forgery. Moreover, by uploading the IC for each specific round to the DFS, $Address_{ic}$ is returned and embedded into $Network_{mc}$, implemented based on the private blockchain HF, and distributed and stored because of the decentralized structure, whereby all $Node_{ic}$ agree for consensus. Thus, decentralization can be guaranteed, and there is no possibility of service failure owing to DDoS attacks and single-point errors.

## 6. Experiment

**Figure 9:**
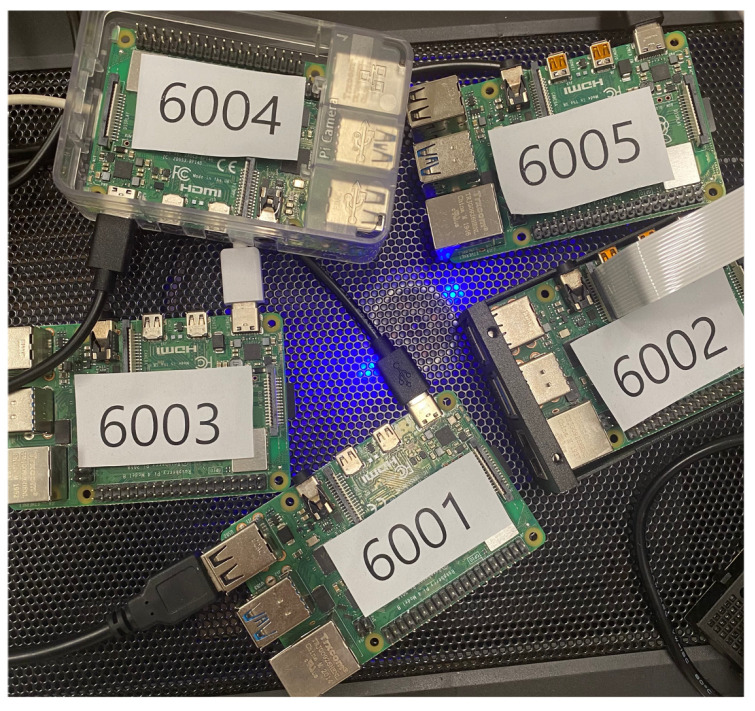
IoT device for IoT-Chain network configuration.

Table 5 is the experimental environments. An experiment was conducted using three Raspberry Pi 4 B computers (system on chip: Broad-com BCM2711, quadcore Cortex-A72 (ARM v8) 64-bit SoC @ 1.5 GHz, memory: 4 GB LPDDR4-3200 SDRAM, OS: Raspbian GNU Linux 10). As depicted in Figure 9, an IoT device network was configured to verify whether the $Network_{ic}$ configuration can be built in an IoT network. Furthermore, by considering the increase in the blockchain size and latency according to the data generated by the actual IoT device, the average file size according to the file format, and the average data size generated by the IoT device for TPS measurement, the experimental data were converted into 8, 128, 1K, and 10K. Decentralization was guaranteed, and there was no possibility of service failure owing to DDoS attacks and single-point errors.

### 6.1. IoT-Chain Size

**Figure 10:**
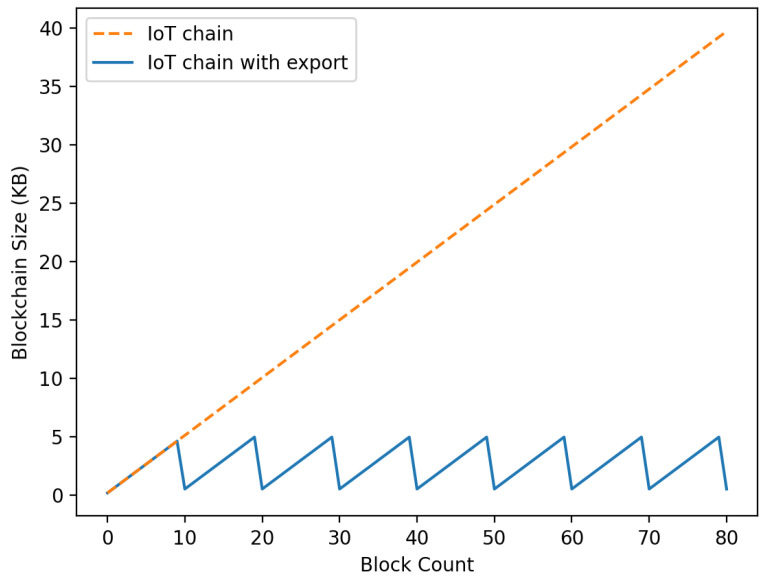
Size comparison according to IoT-Chain export consensus.

In $Network_{iot}$, after consensus is reached for each round in which the IC becomes a specific size, the IC is distributed and stored in the DFS to convert $Address_{ic}$ into $Network_{mc}$. The blockchain size is lightened through the process of sending it, embedding it, and starting the IC again. Depicted in Figure 10 are the size of the IC to which the proposed export was applied and the IC to which the proposed export was not applied. The standard of the round was when the size of the IC became 5 kB. The experiment demonstrated that the size of IC to which the export process was applied did not exceed 5000 kB, but it was confirmed that the size of the blockchain continued to increase when it was not applied.

### 6.2. Consensus Algorithm

**Figure 11:**
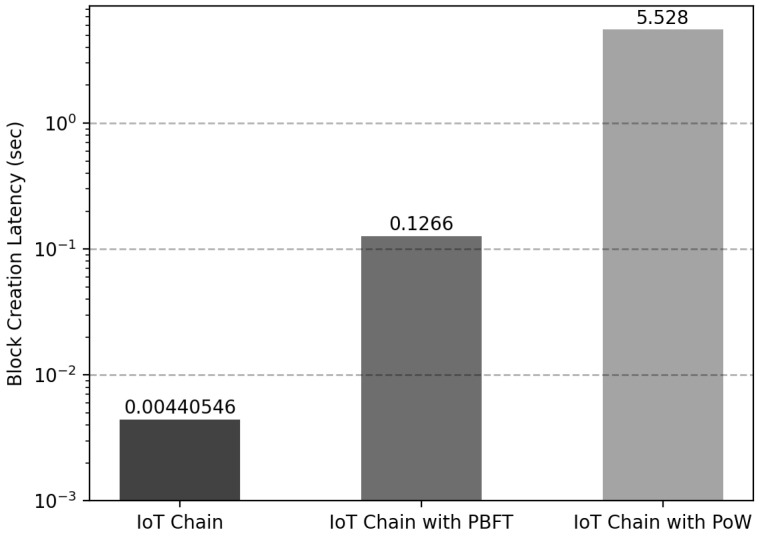
Latency comparison according to IoT-Chain consensus algorithm.

In $Network_{ic}$, the delay time is reduced by randomly selecting $Node_{leader}$ for consensus when creating $Block_{ic}$. As a result, only the delay time corresponding to 0.004 s is displayed. For a performance comparison, the PBFT consensus algorithm implemented in the Fusion-Chain of the IoT-Chain node was applied, and the PoW consensus algorithm with Nonce=2 was applied to compare the delay time. As a result of generating $Block_{ic}$ from $Network_{ic}$ by applying the proposed consensus algorithm, there was an average delay of 0.0044 s, as depicted in Figure 11. However, when blocks were generated through the PBFT consensus algorithm, the average delay time was 0.1266 s. The average delay time of 5.528 s was measured when using PoW. Through the proposed consensus algorithm, the block generation time was reduced by 96% compared to PBFT and 99% compared to PoW. Moreover, when a leader node is selected, a DDoS attack can occur because all nodes can know the leader node when using the method of sending a transaction to the leader node recorded in the last block, but the problem could be solved with a delay of only 0.0044 s. The experimental results confirmed that the latency was reduced compared to the representative consensus algorithm with high CPU computation and network communication.

**Figure 12:**
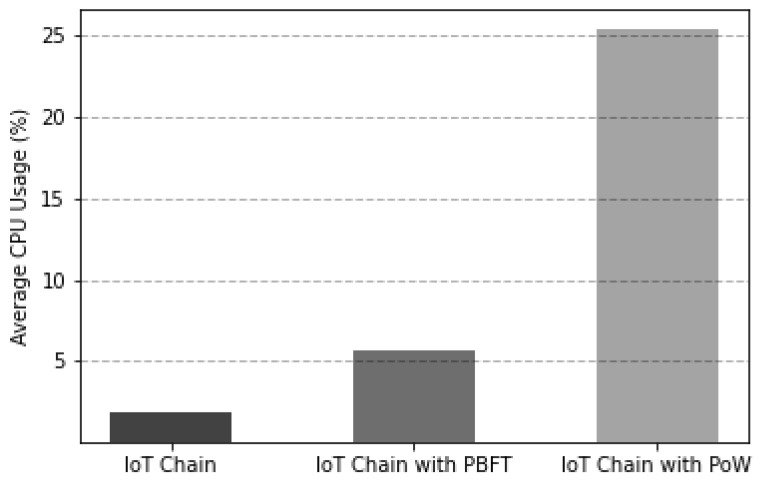
CPU usage comparison according to IoT-Chain consensus algorithm.

As depicted in Figure 12, which is the experimental result of comparing the average CPU usage of the leader node and the consensus participating node during consensus for block generation, it was confirmed that the proposed consensus algorithm had the lowest average CPU usage.

The experiments were conducted by changing the consensus algorithm of IoT-Chain to PoW, PBFT, and the proposed consensus algorithm. In the case of CPU usage, average CPU usage was sampled for 1 s, and the average CPU usage values of the leader node (PBFT, proposed consensus algorithm) and mining node (PoW) were measured from block creation to consensus. The number of nodes of all IoT-Chains was the same, five, and the difficulty nonce value of PoW was set to two.

### 6.3. Block Propagation Delay

The time for the block to propagate from $Node_{leader}$ to $Node_{ic}$ was measured according to the data size to check whether the data size affected the propagation to nodes after the block was created in $Network_{ic}$. To measure the block propagation delay time according to various data sizes in $Network_{ic}$, the data were divided into sizes of 8, 128, 1K, and 10K bytes; and blocks were created in the leader node for five IoT devices, $Node_{ic}$.

For the experiment, after the block was created, the block propagation delay time from $Node_{leader}$ to all $Node_{ic}$ was measured, and the maximum, minimum, and average Boxplot graphs are displayed.

**Figure 13:**
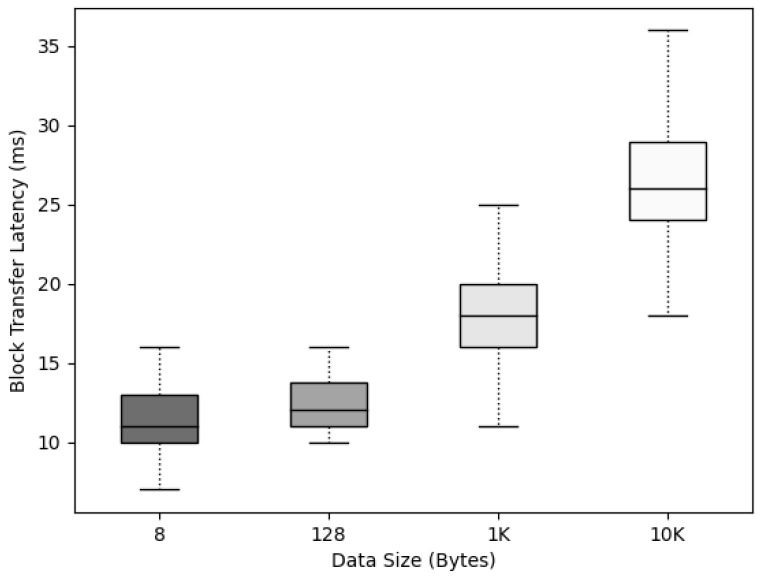
$Block_{ic}$ propagation time according to data size.

By operating and configuring $NetworK_{ic}$, we measured the propagation time after agreeing to $Block_{ic}$, as depicted in Figure 13. It was confirmed that the propagation time of $Block_{ic}$ increased depending on the data size, but the increase was not large, and the maximum average delay time was only 36 ms.

### 6.4. Schnorr Signature

**Figure 14:**
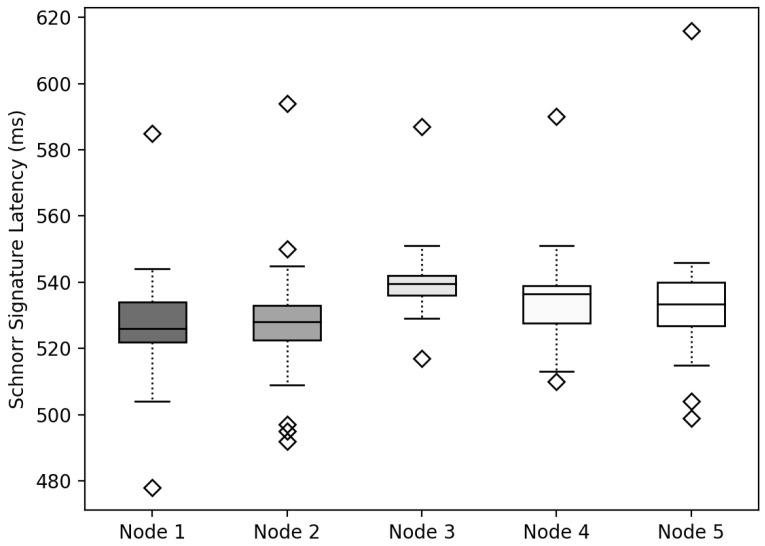
Latency measurement results for generating Schnorr signatures.

To export the IC from $Network_{ic}$ to $Network_{mc}$, Sig was created using the Schnorr signature method to verify that the IC of $Node_{ic}$ was the same as $BH_{last}$. To measure the delay time of the method, the delay time of generating $Sig_{schnorr}$ by combining the signatures generated by encrypting 128 bytes of data with $Key_{priv}$ according to the number of nodes was measured, as depicted in Figure 14. The experimental results revealed that the IoT device generated $Sig_{schnorr}$ while increasing the number of $Node_{ic}$, but the delay time for generating $Sig_{schnorr}$ from each $Node_{ic}$ was constant. It was confirmed that when generating the Schnorr signatures, there was no significant difference in delay time, even if the number of signatures to be combined increased.

### 6.5. Export Latency

**Figure 15:**
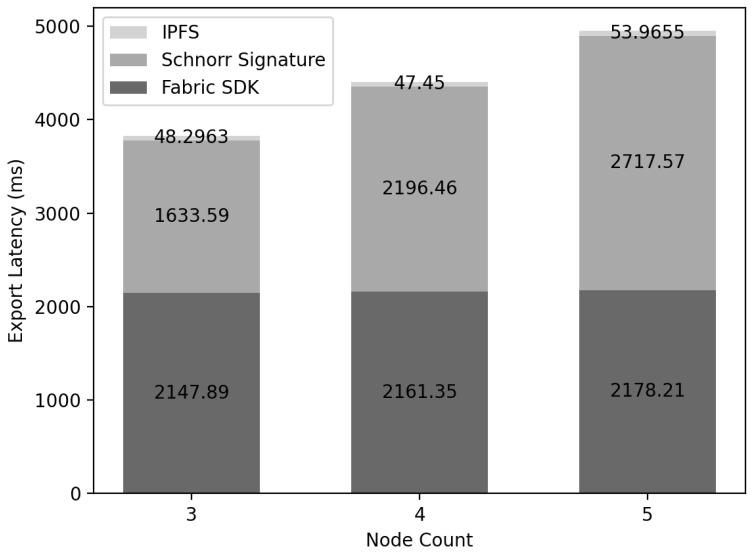
Export latency: the results of measuring the delay time when exporting the IC from $Network_{ic}$ to $Network_{mc}$.

Figure 15 depicts the results of measuring the delay time when exporting the IC from $Network_{ic}$ to $Network_{mc}$. The delay time consisted of three parts: generating the Schnorr signature, uploading the IC to the DFS, and transferring $Network_{ic}$ to $Network_{mc}$$Block_{export}$. The transmission latency consisted of $Network_{ic}$ using the DFS to embed the IC with $Network_{mc}$. $Node_{export}$ uploaded the IC held in the configured DFS by all $Node_{ic}$ that were verified, and it was confirmed that the delay time was only 50 ms. For embedding from $Network_{ic}$ to $Network_{mc}$, all $Node_{ic}$ have the same IC and go through the process of agreeing to export. During this process, all nodes in $Node_{ic}$ must receive and verify Sig. However, since signatures of each node are all generated, there is an O(N) overhead due to signatures in $Node_{ic}$.

In this paper, the signatures of nodes participating in consensus can be combined into one signature through the Schnorr signature. In this way, each node reduces the storage capacity through $Sig_{schnorr}$ compressed with signatures in O(1), and verification is possible at the same time.

As a result of measuring the delay time of the Schnorr signature, it was confirmed that the total delay time of signature verification of $Node_{ic}$ increases linearly in the consensus process because each node verifies the signature O(1) times. Additionally, to insert IC from $Network_{ic}$ to $Network_{mc}$, IC was uploaded to DFS, and the returned address and the combined signature of all nodes were sent to MC, so the final result, the size of the signature stored, is the size of one signature.Fabric SDK can be executed asynchronously with a delay that includes all processes from propagating a transaction to MC(Hyperledger Fabric), consensus and saving it, so it was confirmed that the delay in the export process takes about 2 s.

### 6.6. IoT-Chain Transactions per Second

**Figure 16:**
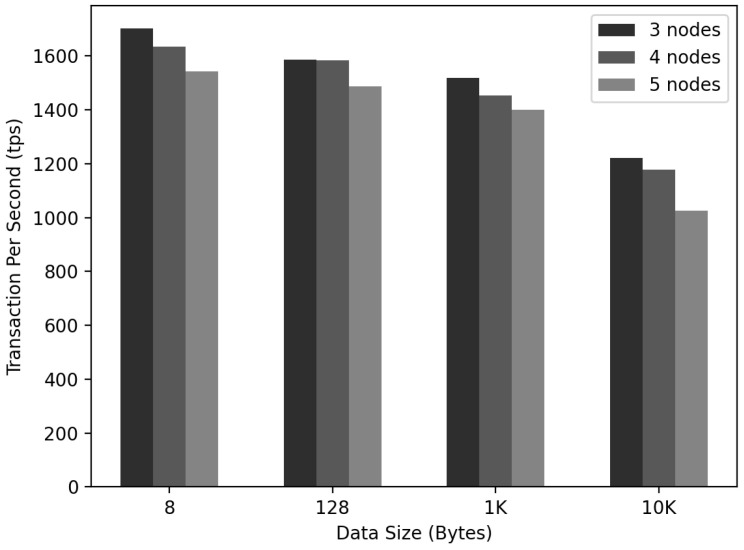
IoT-Chain TPS measurement.

As depicted in Figure 16, an experiment was conducted to confirm the change in the TPS according to the number of $Node_{ic}$ in $Network_{ic}$ and the data size. The size of the experimental data was 8, 128, 1K, or 10K bytes. TX was created, and for the TPS calculation, (1) $Size_{Blockchain}$/$Size_{Tx}$ = $Size_{TxPerBlock}$ and (2) $Size_{TxPerBlock}$/$Time_{BlockCreation}$ = TPS.

(1) and (2) were used to calculate the TPS. When the data size was 8 bytes, the TPS decreased to 1701, 1634, and 1542 as the number of $Node_{ic}$ increased. In the case of 128 bytes, it decreased to 1586, 1583, and 1487. In the case of 1K, it decreased in the order of 1517, 1453, and 1401. At 10K, the TPS decreased to 1220, 1178, and 1024. In all tested cases, the TPS was kept above 1000.

## 7. Conclusions

We have proposed a lightweight blockchain and multilevel blockchain structure for IoT security. The challenges of existing blockchains, such as increasing the blockchain capacity, the consensus algorithm’s calculation amount, the network communication volume, and privacy not being guaranteed, can be overcome through this blockchain structure. To solve the problem of increasing the blockchain capacity, the IC that can be operated in the IoT is stored in the DFS for each specific round, and the returned $Address_{ic}$ is delivered to $Network_{mc}$ and stored to save the IC. To solve the problem of excessive computation and network traffic of the existing consensus algorithm, the VRF-based leader node election method, which is a random function, is used, and the average delay time was only 0.004 s. A stable and tamper-resistant embedding method was applied by constructing a multi-chain architecture and establishing a reliable metadata delivery consensus method and export node between chains (IoT-Chain–Monitoring-Chain). We have proposed a consensus algorithm that is suitable for IoT devices that produce data and generate transactions. It was confirmed that the block propagation time was not affected by the data size. Moreover, in the case of the IC export, when generating Schnorr signatures during the consensus process, the delay time was not affected by the number of nodes. As a result of the total time measurement analysis of the export, only the delay time of the Schnorr signature increased linearly according to the number of nodes. Furthermore, it was confirmed that the upload time to the DFS and the transfer time from $Network_{ic}$ to $Network_{mc}$ were constant. After distributing the IC to solve the privacy problem, $Address_{ic}$ was stored in the MC that was implemented based on the private blockchain HF so that only users who have been granted access to data can access the data. The results of the TPS measurement demonstrated that one of the performance indicators of the blockchain system, $Network_{ic}$, is suited to devices similar to actual IoT devices. As a result of constructing and measuring the TPS, it was proven to maintain more than 1000 tps. In a future study, the Schnorr signature and data transmission delay time from $NetworK_{ic}$ to $Network_{mc}$ will be reduced to decrease the export time, and a study will be conducted on configuring the optimal route of the routing table of $Node_{export}$.

## Figures and Tables

**Table 1:** Comparison of the existing Lightweight blockchain.

	Node Location	TX Throughput	Storage Overhead	CPU Overhead	Access Control	Scalability	Off Chain Storage	Method	Summary/Discussion
Sensor Chain [20]	On IoT Device	-	Low	Low	X	O	X	- Blockchain structure organized by region - PoS Consensus Algorithm - Lightweight by aggregating sensor values stored in the block chain at specific cycles	- A lightweight blockchain framework suitable for IoT devices with low computing power and storage. - IoT Data loss can occur because the data is averaged and summarized.
Richard Dennis, et al. [22]	On IoT Device	-	Low	-	X	O	X	- ’rolling’ method that deletes all blocks older than a preset period	- Propose a temporal “rolling” blockchain which solves the problem of its current exponential growth, instead replacing it with a constant fixed-size blockchain - IoT Data loss occurs by erasing blocks of a certain period.
IOTA [23]	On Server	1000+	High	High	X	O	X	- DAG (Directed Acyclic Graph) based Tangle Consensus Algorithm - PoW Consensus Algorithm	- Tangle algorithm-based cryptocurrency platform for IoT data storage - CPU operation is required using PoW method.
Li Bai, et al. [24]	On Server	-	Low	Mid	O	X	Built in	- Data access control using smart contracts - On-demand data sharing using smart contracts - Off-chain networks to solve storage and data processing challenges	- A light-weighted Blockchain-based platform for IIoT to address security, trust, and island connection problem in the process of IIoT ecosystem construction - Blockchain nodes cannot be operated on-iot device.
Fusion Chain [25]	On IoT Device	-	Low	Low	X	O	Built in	- Distributed storage for storing ’blocks’ - PBFT-based consensus algorithm - PKI method to ensure data privacy	- A decentralized lightweight blockchain that can operate on IoT devices by solving the limitations of blockchain for IoT Security and Privacy - Based on the PBFT consensus algorithm, $O(n^{2})$ is required.
Edge chain [26]	On Server	-	High	Low	O	O	X	- Blockchain-based IoT data management - IoT device regulation using smart contracts - Credit-based Edge Device Resource Management	- Integrates a permissioned blockchain to link the edge cloud resources with each IoT devices account, resource usage and hence behavior of the IoT device - TPS measurement experiment was not carried out.
* Proposed Structure	On IoT Device	1000+	Low	Low	O	O	Built in	- IoT-Chain for IoT device data generation and temporary storage - Monitoring-chain for IoT data monitoring and data access control - Schnorr signature-based data access control and signature lightweight - Data transfer method between blockchain networks using distributed file system - PBFT-based consensus algorithm	- A lightweight multi-level blockchain network consisting only of IoT device node that can ensure IoT security and data privacy

**Table 2:** Notation.

Symbol	Description
IC	IoT-Chain
MC	Monitoring-Chain
HF	Hyperledger Fabric
$Network_{ic}$	Network composed of IoT-Chain nodes
$Network_{mc}$	Hyperledger Fabric-based Monitoring-Chain network
DFS	Distributed file system
$Address_{ic}$	Address returned after uploading IoT-Chain to DFS
Route	Consensus path maintained by export nodes
$Key_{priv}$	Private key for signing IoT-Chain nodes
Sig	Signature of IoT-Chain node
$Key_{pub}$	Public key to verify IoT-Chain node signature
$Sig_{schnorr}$	Combined signatures for consensus upon export
$Key_{schnorr}$	Combined public key to verify consensus during export
CA	Certificate authority for IoT-Chain node registration
Cert	Certificate for public key verification of IoT-Chain node
$Val_{rand}$	Verifiable random value returned after VRF function execution
Round	Restart cycle for IoT-Chain lightweight
SC	Smart contracts installed on the Monitoring-Chain
$Block_{ic}$	Blocks created in IoT-Chain
$Block_{export}$	Address, last block hash signature is stored in block
$Block_{mc}$	Blocks created in Monitoring-Chain
$Block_{genesis}$	Genesis Block when IoT-Chain is restarted
$BH_{last}$	Last block hash value of IoT-Chain
$BH_{genesis}$	Genesis Block hash
$Node_{ic}$	Nodes that create transactions and maintain the blockchain in IoT-Chain
$Node_{mc}$	Node that maintains the blockchain in Monitoring-Chain
$Node_{leader}$	Nodes that are randomly selected from IoT-Chain to generate blocks
$Node_{export}$	Node acting as a gateway in IoT-Chain

**Table 3:** STRIDE Threat Modeling [48].

	Treat	Threat Definition	Property Violated
S	Spoofing Identify	Pretending to be something or someone other than yourself	Authentication
T	Tampering with Data	Modifying something on disk, network, memory, or elsewhere	Integrity
R	Repudiation	Claiming that you didn’ t do something or were not responsible; can be honest or false	Non-repudiation
I	Information Disclosure	Providing information to someone not authorized to access it	Confidentiality
D	Denial of Service	Exhausting resources needed to provide service	Availability
E	Elevation of Privilege	Allowing someone to do something they are not authorized to do	Authorization

**Table 4:** STRIDE threat modeling—results.

Zone	Component	Property Violated	Description
IoT Device	IoT Device /User	S	Authorization can be obtained when a device or user authenticates by disguising as another user.
		E	Attacks on data are possible if the access right of the device or user is allowed.
IoT Field Gateway	IoT Cloud Gateway	S, TRID	Information disclosure and data tampering occur through techniques such as spoofing attacks on gateways that exist outside the IoT network.
	IoT Edge Gateway	S, TRID	
Cloud/Edge Computing	Cloud Eventhub	TRID	- Due to centralized EventHub management, communication interference between gateways and eavesdropping may occur. - Data forgery occurs in storage due to centralized administrators or DDoS attacks.
	Edge Eventhub	TRID	

**Table 5:** Experimental environment.

Type	Name	Function	Specs (Version)
HW	Node in Monitoring Chain	Running Monitoring Chain Node	DellEMC PowerEdge R740 server (CPU: Intel Xeon Silver 4210R 2.4 G, RAM: 32 GB, Ubuntu 18.04)
	Node in IoT Chain	Running IoT Chain Node	Raspberry Pi 4 B (system on chip: Broad-com BCM2711, quadcore Cortex-A72 (ARM v8) 64-bit SoC @ 1.5 GHz, memory: 4 GB LPDDR4-3200 SDRAM, OS: Raspbian GNU Linux 10)
SW	Node.js	Implementation Node in IoT Chain	v14.12.0
	Fabric-client	Connect IoT Chain to Monitoring Chain	v1.4.17
	Hyperledger Fabric	Deploy Node in Monitoring Chain	v2.0
	Golang	Implementation Monitoring Chain Smart Contract	v1.13
	Docker	Deploy Node in Monitoring Chain	v20.10.7

## Data Availability

Not applicable.
